# Supplementary material for: Long-term effects of group rights to fisheries: Evaluating the Western Alaska Community Development Quota program
Source: PLoS One. 2024 Dec 2;19(12):e0312682. doi: 10.1371/journal.pone.0312682 (PMC11611149; doi:10.1371/journal.pone.0312682)
Supplement: S1 File — S1 Appendix. Detailed Statistical Results. (DOCX) [file pone.0312682.s001.docx]

**S1 Appendix. Detailed Statistical Results**

S1 Table shows the definition and summary statistics for all variables included in the statistical equations. The variable means and standard deviations in S1 Table are derived from Census Public Use Microdata Samples (PUMS) of the full set of individual Census records used in the regression equations for the rural Alaska region. The PUMS data shown in S1 Table therefore represent a 5 percent sample of the actual data used to estimate the equations and test hypotheses about the CDQ program. The PUMS samples themselves cannot be used to test hypotheses about the effects of the CDQ program, because their geographic detail is limited to the Census PUMA level.

S2 Table shows the equations for language and educational attainment. The equations were all estimated with year and county fixed effects, which are not shown in the table. The results show, not surprisingly, that age and race are the primary determinants of whether an Indigenous language is spoken at home in rural Alaska communities. The age of the oldest household member is the strongest predictor of household language. Older people are more likely to live in a household where an Indigenous language is spoken, with every year of age adding more than four percent to the relative odds. AIAN individuals living in a household with non-AIAN members, or even a household with individuals reporting mixed AIAN and other identities have much lower odds of speaking an Indigenous language at home. Households with more women or a disabled person are also significantly more likely to use an Indigenous language. Community characteristics also strongly affect the use of Indigenous languages, indicating that language preservation is determined by social as well as a household characteristics. Although Indigenous language use is much lower for comparable households living in the larger hub communities, it is higher in larger villages than smaller villages. The community racial mix shows a quadratic relationship, with language use initially declining as the percentage of the population that is AIAN increases from zero, up to about 25 percent of the population. Above that level, Indigenous language use increases at an increasing rate.

Indigenous language use is much more prevalent in CDQ communities than in communities with comparable demographics and household composition (odds ratio >2, p<.001), both in 1990 and in the period from 2000 onward (coefficients suppressed to protect confidentiality of responses in the relatively small populations). However, the difference between the two periods is not significant. That said, equations estimated with a post-2000 time trend indicate that the language disparity from non-CDQ communities is increasing by over two percent per year (p=.01) (equation cannot be shown due to Census Bureau disclosure rules).

The results for educational attainment, an individual variable, exhibit large gender disparities as well as differences by age and race. After controlling for young adults who are often still in school, possessing a high school degree or equivalent bears a strongly quadratic relationship with age, while individuals born before 1940 (age 50 and older in 1990) are much less likely to have either a high school or college education. Women, especially younger women, are likely to have more education than men, although the disparity diminishes with age. The higher female high school rate is not significant in 1990, but becomes significant after 2000 (p<.001) (equation not shown to protect confidentiality of responses). Individuals with disabilities are much less likely to have either a high school or a college degree. Individuals reporting multiple racial identities are more likely to have both high school and college degrees than those identifying as AIAN alone. Multiracial individuals much more likely to have college degrees after 2000, but there is no significant difference in 1990 (equation not shown to protect confidentiality of responses). At the community level, the AIAN percentage of the population has a strongly quadratic relationship, with AIAN educational attainment reaching a maximum for both high school and college when about 40 percent of the total population is AIAN.

S3 Table displays results for the likelihood that an individual worked at least 40 weeks a year, the natural logarithm of annual earnings, and the natural logarithm of annual income of AIAN individuals age 16 and older living in rural Alaska communities. County and year fixed effects are omitted from the table. Household characteristics as well as individual attributes are presumed to affect labor force participation, which is a prerequisite to year-round employment. Labor force participation also presumably would have a large effect on earnings and income. S3 Table shows that the results largely borne out these assumptions. Signs and significance of individual and household characteristics that affect the probability of working 40 weeks or more are mostly similar for year round employment, earnings, and income. The major exception occurs with gender, where the negative wage disparity largely washes out the higher likelihood of year-round employment. Having an elder in the household also increases income relative to earnings, as would be expected. All three equations show the same quadratic relationship for age, negative effect for women with more young children at home, and large negative effect of disability. Individuals reporting mixed AIAN identities, those living in households with non-AIAN people, and residents of communities with a lower percentage AIAN population all have higher year-round work, earnings, and income, likely signaling greater participation in the local subsistence economy. Year-round employment, earnings, and income are also much higher in regional hub communities, but lower in larger non-hub villages.

Once all the strong demographic characteristics are taken into account, the results show that participation in the CDQ program is associated with a significant, positive effect on year-round employment, earnings, and income, and a significant effect on reducing the likelihood that family income will fall below the poverty threshold.

The results for equations estimated for household weeks worked, earnings, income, and poverty status are shown in S4 Table. These equations represent households, or families in the case of poverty status; consequently, only household demographic variables appear in the equations. Per-capita household earnings, like year-round employment, were significantly lower in CDQ communities in 1990. Per-capita household earnings, however, were significantly higher in CDQ communities from 2000 forward (p<.05), with no trend after 2000. Differences in villages rather than in the larger hub communities drove the large positive change from 1990 (equations not shown to protect confidentiality of responses).

After controlling for demographic factors, per-capita household income was also significantly lower in CDQ communities in 1990, especially in villages (p<.001), and the likelihood that family income was below the poverty threshold was significantly higher (p=.004) (equations not shown to protect confidentiality of responses). In both cases, income and poverty in the CDQ communities returned to levels close to those in non-CDQ communities after 2000 (probability of a difference=.19 for income, 0.47 for poverty), with no trends apparent after 2000 (complete equation results suppressed to protect confidentiality of respondents). For poverty status, the signs of the coefficients are the reverse of those for per-capita household income. Unmarried partners and non-relatives living in the household have a much larger effect on poverty status than they do on earnings and income. That is because household income includes income of these household members, while the Census Bureau does not consider unmarried partners to be part of the family for the poverty calculation.

**S1 Table. Summary Statistics for Variables Included in Regression Equations: American Indian and Alaska Native population of Rural Alaska.^a^** Sources: US Census, 1990, US Census, 2000, and American Community Survey, 2005-2016 Public Use Microdata Samples.

| *Variable name* | *Definition* | Mean | Standard deviation |
| --- | --- | --- | --- |
| *Dependent variables* | |  |  |
| Language | Indigenous language spoken in the household (binary) | 0.597 | 0.491 |
| High School | Completed high school (binary) | 0.542 | 0.498 |
| College | College degree (binary) | 0.089 | 0.284 |
| Weeks worked | Worked 40 or more weeks last year (binary) | 0.356 | 0.479 |
| Earnings | Real annual earnings ($2015) | 31,051 | 116,714 |
| Income | Real income ($2015) | 42,587 | 134,320 |
| HH weeks worked | Anyone in the HH worked 40 or more weeks last year (binary) | 0.727 | 0.445 |
| HH earnings | Real per-capita household earnings ($2015) | 21,430 | 69,138 |
| HH income | Real per-capita household income ($2015) | 28,614 | 81,086 |
| Poverty status | Below poverty threshold (binary) | 0.216 | 0.412 |
| *Explanatory variables* | |  |  |
| *Individual characteristics* | |  |  |
| female | Female gender (binary) | 0.448 | 0.497 |
| age | Age (years) | 42.2 | 16.8 |
| age2 | Age squared | 2060 | 1537 |
| agefemale | Age times female (years) | 19.0 | 24.0 |
| bornbefore1940 | Birth date before 1940 (binary) | 0.049 | 0.217 |
| age16to20 | Age between16 and 20 (binary) | 0.134 | 0.341 |
| age16to23 | Age between16 and 23 (binary) | 0.211 | 0.408 |
| nonrel | Not a relative of the survey respondent | 0.029 | 0.295 |
| multirace | Indicated two or more races (binary) | 0.096 | 0.295 |
| disability | Reported any disability (binary) | 0.124 | 0.330 |
| gq | Group quarters resident | 0.008 | 0.092 |
| *Household characteristics* |  |  |  |
| bornbefore1940hh | Someone in the household born before 1940 (binary) | 0.119 | 0.323 |
| elderhh | Someone in the household aged 60 or older (binary) | 0.250 | 0.433 |
| children | Any children under 18 in the household (binary) | 0.596 | 0.491 |
| kids | Number of children under 18 in the household | 1.63 | 1.82 |
| tots | Number of children under 6 in the household | 0.472 | 0.822 |
| female_tots | Female times number of household children under 6 | 0.022 | 0.195 |
| singlemom2 | Household with children under 6 but no adult males (binary) | 0.005 | 0.067 |
| nonaianhh | Any non-AIAN person in the household (binary) | 0.112 | 0.315 |
| hh_hs | Number of household high school graduates | 2.533 | 1.575 |
| hh_college | Number of household college graduates | 0.514 | 0.735 |
| hh_adults | Number of household members age 16 and older | 3.03 | 1.52 |
| hh_female | Number of household females over age 18 | 1.47 | 0.93 |
| hh_pcths | Fraction of household members 16 and older with high school diploma | 0.870 | 0.411 |
| hh_pctcollege | Fraction of household members 16 and older with a college degree | 0.183 | 0.288 |
| hh_pctadults | Fraction of household members 16 and older | 0.664 | 0.244 |
| hh_pctfemale | Fraction of household members 16 and older who are female | 0.488 | 0.235 |
| hh_age | Age of oldest household member | 50.8 | 15.3 |
| hh_age1 | Average age of household adults | 39.4 | 11.7 |
| hh_female_age1 | Average age of household female adults | 36.4 | 17.2 |
| multiracehh | At least one member of the household identifying as AIAN and another race (binary) | 0.142 | 0.349 |
| hh_age16to20 | Fraction of household members aged 16 and older who are under 21 | 0.551 | 0.840 |
| all_ya | All household members are aged 21-39 (binary) | 0.022 | 0.148 |
| hhdisable | Any household member has a disability (binary) | 0.390 | 0.488 |
| *Community characteristics* | |  |  |
| CDQ community | CDQ-eligible community (binary) | 0.362 | 0.481 |
| totpop | Community population | 476.8 | 1002.8 |
| logpop | Natural logarithm of community population | 5.026 | 3.916\ |
| pctaian | AIAN fraction of community population | 0.656 | 0.475 |
| pctaian2 | Square of AIAN fraction of population | 0.558 | 0.497 |
| hub | Population 2,000 or greater (binary) | 0.231 | 0.421 |

^a^ Rural Alaska defined as Public Use Microdata Area (PUMA) 300 (1990), or Alaska PUMA 400 (2000, 2005-2016).

**S2 Table. Logistic regression equations for Indigenous language and educational attainment**

(weighted maximum likelihood estimates, year and county fixed effects not shown)

|  | **Household language** | | | **High school degree** | | | **College degree** | | |
| --- | --- | --- | --- | --- | --- | --- | --- | --- | --- |
| Variable name | Coef. | z |  | Coef. | z |  | Coef. | z |  |
| CDQ community^a^ | 0.044 | 0.39 |  | -0.122 | -1.69 | * | -0.061 | -0.30 |  |
| female |  |  |  | 0.304 | 3.81 | *** | 1.22 | 3.69 | *** |
| hh_female | 0.192 | 4.84 | *** |  |  |  |  |  |  |
| age |  |  |  | 0.152 | 20.8 | *** | 0.085 | 4.06 | *** |
| age2 |  |  |  | -0.00179 | -22.0 | *** | -0.000534 | -2.37 | ** |
| agefemale |  |  |  | -0.00531 | -2.90 | *** | -0.0129 | -1.91 | * |
| hh_age | 0.045 | 10.20 | *** |  |  |  |  |  |  |
| hh_age1 | -0.008 | -1.16 |  |  |  |  |  |  |  |
| bornbefore1940 | 0.527 | 3.31 | *** | -0.736 | -9.32 | *** | -1.15 | -4.43 | *** |
| bornbefore1940hh | -0.207 | -1.70 | * |  |  |  |  |  |  |
| age16to20 |  |  |  | -1.66 | -24.6 | *** |  |  |  |
| age16to23 |  |  |  |  |  |  | -2.85 | -7.24 | *** |
| multirace |  |  |  | 0.312 | 4.02 | *** | 1.11 | 8.68 | *** |
| multiracehh | -0.346 | -3.39 | *** |  |  |  |  |  |  |
| disability |  |  |  | -0.454 | -9.61 | *** | -0.673 | -5.62 | *** |
| hhdisable | 0.367 | 5.57 | *** |  |  |  |  |  |  |
| elderhh | 0.151 | 1.50 |  |  |  |  |  |  |  |
| all_ya | -0.233 | -1.03 |  |  |  |  |  |  |  |
| nonaianhh | -0.635 | -4.74 | *** |  |  |  |  |  |  |
| totpop |  |  |  | -8.20E-06 | -1.36 |  | 8.83E-006 | 2.20 | ** |
| totpop2 |  |  |  | 8.95E-11 | 1.42 |  |  |  |  |
| logpop | 0.424 | 8.95 | *** |  |  |  |  |  |  |
| pctaian | -2.26 | -2.33 | ** | 1.28 | 2.31 | ** | 2.20 | 1.90 | * |
| pctaian2 | 4.39 | 6.19 | *** | -1.54 | -3.61 | *** | -2.53 | -2.82 | *** |
| hub | -0.568 | -4.21 | *** | 0.211 | 1.54 |  | 0.0993 | 0.26 |  |

| Observations | 103,000 | 65,500 | 65,500 |
| --- | --- | --- | --- |
| Wald statistic | 3,518*** | -- | 763.8*** |
| Wald df | 48 | 46 | 46 |
| Pseudo R^2^ | 0.373 | 0.209 | 0.147 |
| Log likelihood | -370,100 | -283,500 | -74,260 |

^a^ Coefficient represents difference between estimate for years 2000-2016 and estimate for 1990.

*p <.1, **p<.05, ***p<<.01

**S3 Table. Logistic and censored regression equations for employment, earnings, and income**

(weighted maximum likelihood estimates, year and county fixed effects not shown)

|  | **Worked 40+ weeks** (logistic regression) | | | | **Log of annual earnings** (censored regression) | | | | **Log of annual income** (censored regression) | | |  |
| --- | --- | --- | --- | --- | --- | --- | --- | --- | --- | --- | --- | --- |
| Variable name | Coef. | z |  | | Coef. | z |  | | Coef. | z |  |  |
| CDQ community^a^ | 0.160 | 2.00 | ** | | 0.385 | 2.30 | ** | | 0.108 | 2.21 | ** |  |
| female | 0.576 | 5.16 | *** | | 0.207 | 0.93 |  | | -0.0306 | -0.54 |  |  |
| age | 0.171 | 19.82 | *** | | 0.530 | 27.72 | *** | | 0.0464 | 11.59 | *** |  |
| age2 | -0.00180 | -17.98 | *** | | -0.00636 | -27.79 | *** | | -2.02E-04 | -4.45 | *** |  |
| agefemale | -0.0128 | -5.14 | *** | | -0.0349 | -6.45 | *** | | -0.0046 | -3.98 | *** |  |
| disability | -0.645 | -10.14 | *** | | -2.937 | -17.92 | *** | | -0.253 | -6.91 | *** |  |
| children | 0.0752 | 1.44 |  | | 0.135 | 1.19 |  | | 0.0307 | 0.74 |  |  |
| kids | -0.0567 | -3.59 | *** | | -0.203 | -6.07 | *** | | -0.00883 | -0.70 |  |  |
| female_tots | -0.173 | -4.31 | *** | | -0.389 | -4.32 | *** | | -0.118 | -3.14 | *** |  |
| bornbefore1940 | -0.316 | -2.72 | *** | | -1.35 | -4.51 | *** | | -0.0451 | -0.72 |  |  |
| elderhh | 0.122 | 1.91 | * | | -0.215 | -1.39 |  | | 0.145 | 3.79 | *** |  |
| nonaianhh | 0.285 | 4.39 | *** | | 0.690 | 4.74 | *** | | 0.172 | 3.44 | *** |  |
| multirace | 0.238 | 3.35 | *** | | 0.838 | 5.44 | *** | | 0.174 | 3.23 | *** |  |
| age16to20 | -1.19 | -11.01 | *** | | -2.16 | -11.61 | *** | | -0.959 | -19.11 | *** |  |
| nonrel | -0.360 | -5.37 | *** | | -0.636 | -4.71 | *** | | -0.311 | -6.94 | *** |  |
| gq | -0.521 | -3.09 | *** | | -1.01 | -2.62 | *** | | -0.868 | -5.72 | *** |  |
| hh_adults | -0.146 | -6.51 | *** | | -0.324 | -6.28 | *** | | -0.125 | -9.15 | *** |  |
| hh_female | 0.146 | 5.03 | *** | | 0.277 | 4.20 | *** | | 0.0892 | 4.66 | *** |  |
| hh_age | -0.0212 | -5.97 | *** | | -0.0489 | -6.57 | *** | | -0.0153 | -7.23 | *** |  |
| hh_age1 | -0.0105 | -2.66 | *** | | -0.0184 | -1.91 | * | | -0.0100 | -4.86 | *** |  |
| hh_female_age1 | 0.00544 | 4.60 | *** | | 0.0177 | 6.01 | *** | | 0.00338 | 4.81 | *** |  |
| bornbefore1940hh | 0.198 | 2.49 | *** | | 0.364 | 1.99 | ** | | 0.0853 | 1.61 |  |  |
| hh_age16to20 | 0.0736 | 2.23 | ** | | 0.395 | 5.35 | *** | | 0.114 | 5.84 | *** |  |
| hhdisable | -0.138 | -2.92 | *** | | -0.102 | -0.91 |  | | -0.0547 | -1.78 | * |  |
| totpop | -1.34E-06 | -0.65 |  | |  |  |  | |  |  |  |  |
| logpop |  |  |  | | -0.269 | -4.55 | *** | | -0.0322 | -1.90 | * |  |
| pctaian | -1.162 | -7.55 | *** | | -2.55 | -8.01 | *** | | -0.861 | -8.82 | *** |  |
| hub | 0.407 | 4.03 | *** | | 0.541 | 2.98 | *** | | 0.0898 | 1.73 | * |  |
| Observations | | 65,500 | | | 65,500 | | | | 65,500 | | | |
| Wald statistic | | 2756*** | | | 140.5*** | | | | 105.4*** | | | |
| Wald df | | 60 | | | 60 | | | | 60 | | | |
| Pseudo R2 | | 0.149 | | | 0.063 | | | | 0.047 | | | |
| Log likelihood | | -312,600 | | | -1,411,000 | | | | -1,096,000 | | | |

^a^ Coefficient represents difference between estimate for years 2000-2016 and estimate for 1990.

*p <.1, **p<.05, ***p<.01

**S4 Table. Regression equations for household employment, earnings, income, and poverty**

(weighted maximum likelihood or weighted least squares estimates; standard errors, Wald and F tests adjusted for duplicated observations; year and county fixed effects not shown)

|  | | **Anyone in the HH worked 40+ weeks** (logistic regression) | | | **Log of per-capita HH annual earnings** (censored regression) | | | | | **Log of per-capita HH annual income** (OLS regression) | | | **Poverty status** (logistic regression) | | |
| --- | --- | --- | --- | --- | --- | --- | --- | --- | --- | --- | --- | --- | --- | --- | --- |
| Variable name | | Coef. | z |  | Coef. | | z |  | | Coef. | z |  | Coef. | z |  |
| CDQ community | | 0.153 | 1.53 |  | 0.327 | | 2.79 | *** | | 0.150 | 3.73 | *** | -0.202 | -2.02 | ** |
| hh_adults | | 0.283 | 8.75 | *** |  | |  |  | |  |  |  |  |  |  |
| hh_female | | 0.133 | 3.32 | *** |  | |  |  | |  |  |  |  |  |  |
| hh_pctadults | |  |  |  | 0.681 | | 3.18 | *** | | 1.290 | 24.6 | *** | -1.43 | -3.36 | *** |
| hh_pctfemale | |  |  |  | -0.911 | | -5.21 | *** | | -0.0426 | -0.90 |  | 0.269 | 1.04 |  |
| hh_age | | -0.00109 | -0.25 |  | 0.0529 | | 10.64 | *** | | -0.00510 | -3.59 | *** | -0.00173 | -5.26 | *** |
| hh_age1 | | -0.0231 | -4.58 | *** | -0.129 | | -18.95 | *** | | 0.00770 | 5.09 | *** | 0.0105 | 4.14 | *** |
| hh_female_age1 | | 0.00797 | 4.54 | *** | 0.0246 | | 8.07 | *** | | 0.00385 | 5.39 | *** | -0.0179 | -4.21 | *** |
| children | | 0.333 | 4.61 | *** | 0.304 | | 3.72 | *** | | 0.213 | 8.65 | *** | -0.748 | -4.86 | *** |
| tots | | -0.181 | -4.84 | *** | -0.219 | | -7.00 | *** | | -0.0882 | -7.21 | *** | 0.210 | 2.41 | *** |
| kids | | 0.0269 | 1.33 |  | -0.0345 | | -1.65 | * | | -0.0315 | -4.42 | *** | 0.0577 | 2.29 | ** |
| singlemom1hh | | -0.413 | -1.98 | ** | -0.717 | | -1.74 | * | | -0.162 | -2.28 | ** | 0.581 | 1.35 |  |
| singlemom2hh | | -0.578 | -2.29 | ** | -1.01 | | -2.25 | ** | | -0.312 | -3.38 | *** | 0.468 | 1.44 |  |
| bornbefore1940hh | | -0.275 | -3.19 | *** | -0.806 | | -5.62 | *** | | -0.0502 | -1.62 |  | -0.219 | 4.05 | *** |
| elderhh | | -0.338 | -3.98 | *** | -0.907 | | -7.18 | *** | | 0.0264 | 0.81 |  | -0.414 | 0.33 |  |
| nonaianhh | | 0.803 | 7.65 | *** | 0.832 | | 8.03 | *** | | 0.333 | 10.65 | *** | -0.443 | -3.88 | *** |
| multiracehh | | 0.279 | 3.40 | *** | 0.300 | | 3.30 | *** | | 0.144 | 5.00 | *** | -0.370 | 0.88 |  |
| hh_age16to20 | | -0.383 | -10.1 | *** | -0.571 | | -13.66 | *** | | -0.0762 | -5.94 | *** | 0.0178 | 2.06 | ** |
| hhdisable | | -0.488 | -9.33 | *** | -0.806 | | -9.82 | *** | | -0.172 | -9.24 | *** | 0.209 | 4.25 | *** |
| partnerhh | | -0.201 | -2.29 | *** | -0.202 | | -2.66 | *** | | -0.108 | -3.75 | *** | 0.859 | 3.25 | *** |
| nonrels | | -0.004 | -0.09 | *** | 0.0294 | | 0.79 |  | | -0.0229 | -1.59 |  | 0.349 | 6.18 | *** |
| gq | | -0.845 | -2.59 | *** | -1.164 | | -1.94 | * | | -0.966 | -3.30 | *** | 1.23 | 6.38 | *** |
| pctaian | | -1.05 | -4.32 | *** | -1.138 | | -3.94 | *** | | -0.628 | -5.98 | *** | 0.951 | 2.99 | *** |
| totpop | | -3.42E-06 | -1.08 |  |  | |  |  | |  |  |  | -5.00E-07 | 0.97 |  |
| logpop | |  |  |  | -0.0483 | | -1.00 |  | | 1.70E-03 | 0.11 |  |  |  |  |
| hub | | 0.634 | 3.79 | *** | 0.281 | | 1.95 | ** | | 0.186 | 3.95 | *** | -0.491 | -2.99 | *** |
| Observations | 104,000 | | | | | 104,000 | | | 104,000 | | | | 103,000 | | |
| Wald or F statistic | 1474*** | | | | | 37.29*** | | | 99.19*** | | | | 1676*** | | |
| Wald or F df | 56 | | | | | 56 | | | 56,104000 | | | | 56 | | |
| R2 or pseudo R2 | 0.117 | | | | | 0.049 | | | 0.297 | | | | 0.130 | | |
| Log likelihood | -509,000 | | | | | -2,006,000 | | | 0.810^b^ | | | | -433,300 | | |

^a^ Coefficient represents difference between estimate for years 2000-2016 and estimate for 1990.

^b^ Root mean-square error

*p <.1, **p<.05, ***p<.01
