# Supplementary material for: Long-term effects of group rights to fisheries: Evaluating the Western Alaska Community Development Quota program
Source: PLoS One. 2024 Dec 2;19(12):e0312682. doi: 10.1371/journal.pone.0312682 (PMC11611149; doi:10.1371/journal.pone.0312682)
Supplement: Attachment — Submitted filename: CDQ review comments.docx [file pone.0312682.s003.docx]

PONE-D-24-19040

Long-term effects of group rights to fisheries: Evaluating the Western Alaska Community Development Quota program

PLOS ONE

Response to Reviewer Comments:

1. The paper should explicitly mention that well-being, especially Indigenous well-being, is multidimensional and a community- and culturally- defined concept.

This understanding is now included in the first paragraph of the methods section (see next comment).

The paper mentions drawing on a definition of well-being provided by the Arctic Human Development Report without stating what that definition is, or that it may not be universally accepted or adopted by a region as diverse as western Alaska. It’s important also to mention that this analysis is limited in scope to social and economic change or benefits that can be systematically measured across communities and thus many important dimensions of well-being and culture remain unaccounted for in the study. This will help to frame or make more acceptable to the reader the selection of indicators as described later on in the paper, for example, the single indicator of Indigenous language retention for cultural continuity.

The first two paragraphs of the methods section mention this issue. This section was revised and expanded to acknowledge more clearly the limitation raised by the reviewer, and inherent in any quantitative evaluation of the CDQ program.

2. Language retention indicator: This single indicator is somewhat problematic as a measure of cultural continuity given the high level of Indigenous language loss that has occurred across Alaska. Many studies suggest that the continuation of traditional hunting and fishing practices and related food practices is vital to Indigenous well-being and a central facet of cultural continuity. The paper states that they don’t have access to good data to bring subsistence practices and foods into the analysis but I would encourage the authors to consider adding at the very least a paragraph in the ‘BS fisheries and communities’ section that describes the mixed-economies of western Alaska and the high reliance and value placed on subsistence practices and foods.

Several sentences were added to the Bering Sea fisheries and communities section to describe the mixed economy of the region and the importance of subsistence practices. The potential interaction of subsistence and small-scale commercial fishing as well as the uneven level of language loss is also mentioned in the hypotheses section on language and cultural continuity. This section also notes that language retention is not an objective of the CDQ program. Whether the CDQ program indirectly affects language retention is a hypothesis to be tested. What is relevant for the study is not the level of language loss, but rather the relative rate of loss since 1990.

3. As currently written it’s unclear how the CDQ program benefits or supports language retention or immersion programs? The authors provide two examples (lines 212-214) but they seem tenuous or perhaps assumed and I’m not sure if that was the intention. To my knowledge, it is primarily Alaska Native Corporations and Tribes that support/lead the efforts to restore Indigenous languages in western AK. If CDQs are involved in language restoration initiatives, providing a few examples to better make the linkage would be helpful.

The potential interaction of subsistence and small-scale commercial fishing as well as the uneven level of language loss is also mentioned in the hypotheses section on language and cultural continuity. This section also notes that language retention is not an objective of the CDQ program. As noted above whether the CDQ program indirectly affects language retention is a hypothesis to be tested. It is not claimed as a fact.

4. Figures were not included in the PDF to review so this may not be an issue but a map of the CDQ region/regions would be really helpful to the reader, especially if the map included where communities are located so the reader can see communities included/excluded from the program. The paper references nearby ineligible communities in the same county, but not a lot of detail about this subset; a map or a list of CDQ and non-CDQ communities included in the analysis may be helpful.

A new figure was created that attempts to show the location of CDQ communities and comparison communities more transparently. There are too many communities (over 150) for a list of communities to be helpful for the reader.

5. Individual earnings and income: This paper mentions that larger economic changes were controlled for in the analysis but it was unclear if and how engagement in non-CDQ commercial fisheries may have shaped results related to earnings and income in CDQ regions. Is there a way to tease these factors apart? For example, I was thinking about the role of state managed salmon, crab, or other (non-CDQ) fisheries and the economic engine they serve in many coastal (CDQ) communities. Oftentimes these communities reap greater benefits than those further upriver or inland because of greater access to commercial fisheries, processing, infrastructure, and ancillary services. These wouldn’t necessarily be CDQ related benefits. Is there a way to control for participation in local fisheries as a contributor to large income benefits or to better account for it in the results section?

The potential for changes in fishery participation outside the CDQ program to affect the results is an important point. It is now addressed in a new paragraph in the discussion section addressing changes in direct fishery participation and harvests. The paragraph includes a new figure that illustrates how fisheries participation has changed over time in CDQ vs. comparison communities. co

6. Most Tribes and Native corporations offer scholarships that would be available to non-CDQ students so that may help to explain the lack of difference in educational outcomes between the two sets of communities if focusing specifically on Alaska Native households.

This was noted and the discussion section and added as another explanation for the finding of no significant difference between the two communities.

What surprises me most about these results is the data related to changes in poverty levels. Findings suggest that poverty rates in CDQ communities have dropped since 1990 but they remain equal to rates in non-CDQ communities since 2000. Is that correct? This is surprising given that one of the main missions of the CDQ program is to alleviate poverty.

Yes, the finding is that poverty rates in CDQ communities have improved, but only to match poverty rates of non-CDQ communities, which were relatively less poor in 1990. In the discussion section, a sentence was added that notes that while the CDQ program did alleviate poverty, these communities started at a disadvantage relative to the comparison communities, and the magnitude of poverty alleviation has only been sufficient to raise these communities to the point of equality with non-CDQ communities.

Other comments:

Line 40 – missing word at end of sentence?

Missing word restored.

Lines 45-47 - The authors should consider reviewing Paul Foley’s work on community rights in Canada. He has many recent papers that should be of interest but I’ve listed two here from 2015 that are especially relevant:

- Governing enclosure for coastal communities: Social embeddedness in a Canadian shrimp fishery (2015), Marine Policy
- Making Space for Community Use Rights: Insights From “Community Economies” in Newfoundland and Labrador (2015), Society and Natural Resources

Thank you for the reminder. A sentence was added mentioning community use rights in the shrimp fishery with citations to Foley. The two articles about community resource rights are relevant, but his other papers are tangential.

Lines 153-4 – should cite Courtney Lyons work here.

- Alaska's community development quota program: A complex institution affecting rural communities in disparate ways (2019), Marine Policy

Citation added in the appropriate place.

In the Intro or Methods section it should be made clear that the CDQ program doesn’t provide actual fishing opportunity for residents of the region outside of the halibut fishery.

This was noted in a sentence added in the section of the Introduction describing the CDQ program.

Add limitations

Limitations were described in the manuscript in several places, but not formally. A new paragraph was added to the discussion section that compiles and describes the aforementioned various study limitations in one place.
