# Supplementary material for: Long-term effects of group rights to fisheries: Evaluating the Western Alaska Community Development Quota program
Source: PLoS One. 2024 Dec 2;19(12):e0312682. doi: 10.1371/journal.pone.0312682 (PMC11611149; doi:10.1371/journal.pone.0312682)
Supplement: Attachment — Submitted filename: Reviewer Comments on PONE.docx [file pone.0312682.s002.docx]

Reviewer Comments on PONE-D-24-19040

Title: Long-term effects of group rights to fisheries: Evaluating the Western Alaska

Community Development Quota program

This is a well-written and structured article, but I think it can be improved upon in a few ways. Most of my suggestions have to do with appropriate framing and further unpacking of results.

Firstly, I think the paper should explicitly mention that well-being, especially Indigenous well-being, is multidimensional and a community- and culturally- defined concept.

The paper mentions drawing on a definition of well-being provided by the Arctic Human Development Report without stating what that definition is, or that it may not be universally accepted or adopted by a region as diverse as western Alaska. It’s important also to mention that this analysis is limited in scope to social and economic change or benefits that can be systematically measured across communities and thus many important dimensions of well-being and culture remain unaccounted for in the study. This will help to frame or make more acceptable to the reader the selection of indicators as described later on in the paper, for example, the single indicator of Indigenous language retention for cultural continuity.

Language retention indicator: This single indicator is somewhat problematic as a measure of cultural continuity given the high level of Indigenous language loss that has occurred across Alaska. Many studies suggest that the continuation of traditional hunting and fishing practices and related food practices is vital to Indigenous well-being and a central facet of cultural continuity. The paper states that they don’t have access to good data to bring subsistence practices and foods into the analysis but I would encourage the authors to consider adding at the very least a paragraph in the ‘BS fisheries and communities’ section that describes the mixed-economies of western Alaska and the high reliance and value placed on subsistence practices and foods.

As currently written it’s unclear how the CDQ program benefits or supports language retention or immersion programs? The authors provide two examples (lines 212-214) but they seem tenuous or perhaps assumed and I’m not sure if that was the intention. To my knowledge, it is primarily Alaska Native Corporations and Tribes that support/lead the efforts to restore Indigenous languages in western AK. If CDQs are involved in language restoration initiatives, providing a few examples to better make the linkage would be helpful.

Figures were not included in the PDF to review so this may not be an issue but

a map of the CDQ region/regions would be really helpful to the reader, especially if the map included where communities are located so the reader can see communities included/excluded from the program. The paper references nearby ineligible communities in the same county, but not a lot of detail about this subset; a map or a list of CDQ and non-CDQ communities included in the analysis may be helpful.

Individual earnings and income:

This paper mentions that larger economic changes were controlled for in the analysis but it was unclear if and how engagement in non-CDQ commercial fisheries may have shaped results related to earnings and income in CDQ regions. Is there a way to tease these factors apart? For example, I was thinking about the role of state managed salmon, crab, or other (non-CDQ) fisheries and the economic engine they serve in many coastal (CDQ) communities. Oftentimes these communities reap greater benefits than those further upriver or inland because of greater access to commercial fisheries, processing, infrastructure, and ancillary services. These wouldn’t necessarily be CDQ related benefits. Is there a way to control for participation in local fisheries as a contributor to large income benefits or to better account for it in the results section?

Finally, most Tribes and Native corporations offer scholarships that would be available to non-CDQ students so that may help to explain the lack of difference in educational outcomes between the two sets of communities if focusing specifically on Alaska Native households.

What surprises me most about these results is the data related to changes in poverty levels. Findings suggest that poverty rates in CDQ communities have dropped since 1990 but they remain equal to rates in non-CDQ communities since 2000. Is that correct? This is surprising given that one of the main missions of the CDQ program is to alleviate poverty.

Other comments:

Line 40 – missing word at end of sentence?

Lines 45-47 - The authors should consider reviewing Paul Foley’s work on community rights in Canada. He has many recent papers that should be of interest but I’ve listed two here from 2015 that are especially relevant:

- Governing enclosure for coastal communities: Social embeddedness in a Canadian shrimp fishery (2015), Marine Policy
- Making Space for Community Use Rights: Insights From “Community Economies” in Newfoundland and Labrador (2015), Society and Natural Resources

Lines 153-4 – should cite Courtney Lyons work here.

- Alaska's community development quota program: A complex institution affecting rural communities in disparate ways (2019), Marine Policy

In the Intro or Methods section it should be made clear that the CDQ program doesn’t provide actual fishing opportunity for residents of the region outside of the halibut fishery.
